# Genicular nerve radiofrequency ablation for pain control following anterior cruciate ligament reconstruction - A case report

**DOI:** 10.1016/j.tcr.2022.100661

**Published:** 2022-05-24

**Authors:** R. Deviandri, V. Yuliana, D. Irawan, A.N. Rahman

**Affiliations:** a Department of Orthopedics, University of Groningen, University Medical Center Groningen, Groningen, the Netherlands; b Department of Physiology, Faculty of Medicine, Universitas Riau, Division of Orthopaedic, Arifin Achmad Hospital, Pekanbaru, Indonesia; c Department of Sport and Rehabilitation, Fit Centrum, Pekanbaru, Indonesia; d Department of Anesthesia, Arifin Achmad Hospital, Pekanbaru, Indonesia; e Department of Orthopedic and Pain Intervention, Sentra Medika Hospital, Cisalak, Indonesia; f Faculty of Medicine, Universitas Padjadjaran, Bandung, Indonesia

**Keywords:** Radiofrequency, ACL tear, Pain intervention, Pain management, Visual Analogue Scale score

## Abstract

Adequate postoperative pain control is an essential factor for the success of rehabilitation programs after anterior cruciate ligament reconstruction (ACLR). The genicular nerve radiofrequency ablation (GNRF) is a recently developed method.

This study aimed to evaluate the use of GNRF in patients who underwent ACLR.

We performed GNRF guided by ultrasonography for patients who underwent ACLR with aggravated pain. GNRF was performed following ACLR a day after surgery. The pain was evaluated using the Visual Analogue Scale (VAS), and the Euroqol-5 Dimension (EQ-5D) measured the quality of life on the day of one, three, and seven after surgery.

**Results:**

In this study, two patients participated. In the postoperative days first, third, and seventh, the mean VAS was decreased after providing the intervention (from 8 to 5, 2, and 1, respectively). The mean EQ5D improved from 0.48 to 0.52, 0.56, 0.66, respectively.

We concluded that GNRF is an adequate and safe procedure for managing postoperative pain after ACLR. It may enhance the postoperative rehabilitation program.

## Introduction

Anterior cruciate ligament (ACL) tear is a common sports injury. The overall age- and sex-adjusted annual incidence of ACL tears was 74.6 per 100,000 person-years. Anterior cruciate ligament reconstruction (ACLR) surgery is frequently performed by orthopedic surgeons worldwide. It is estimated that approximately 400,000–500,000 cases of ACLR are carried out each year in the United States (US) based on implant usage [1].

Recently, ACLR techniques advance with various graft utilization and deeper insight of graft biomechanics. Hamstring tendon, patellar tendon, quadriceps tendon, peroneus longus tendon, and also allograft could be used as a choice of graft in ACLR with comparable biomechanics and outcome [2]. Recent study showed that the biomechanics of various grafts more influenced by the viscoelastic property of the graft itself rather than the preparation method [3].

The success rates of ACLR are high, which is more than 95%. However, the pain remains the most common postoperative complication delaying patient discharge and increasing the costs associated with patient care [4]. ACLR has subjectively been ranked as the most painful procedure compared with other orthopedics sports medicine treatments, including meniscal debridement, rotator cuff repair, and labral repair [5].

Since successful postoperative pain management is often correlated with improved patient outcomes, developing an effective strategy to provide adequate pain relief is needed [6]. Common agents in current practice include narcotics, non-steroidal anti-inflammatory agents (NSAIDs), and anesthetic agents such as lidocaine or bupivacaine with nerve blocking and regional anesthetic capabilities. Less commonly used agents include ketamine, tranexamic acid (TXA), sedatives, gabapentinoids, and corticosteroids [7].

Radiofrequency therapy is a medical procedure mainly used to reduce pain with a low complication rate (less than 1%), ease of application, and low cost (1). The electric current generated by radio waves is used to heat a small nerve tissue area, thereby inhibiting or reducing pain signals from that particular area. Radiofrequency has been developed for diseases related to the spinal functional unit in the few years [8].

Successful management of knee pain by radiofrequency (RF) has been shown [9], [10]. The genicular nerve radiofrequency ablation (GNRF), including conventional, cooled, and pulsed radiofrequency, has been used to manage chronic knee pain, osteoarthritis, and post total knee arthroplasty procedure [9], [10], [11], [12]. However, to the author's knowledge, there is no literature describing the use of GNRF for pain control following ACLR.

The purpose of this study is to evaluate the use of GNRF in patients who underwent ACLR and describe the role of GNRF in practice as a part of a multimodal regimen in pain management after ACLR.

## Patient and method

In this prospective study, GNRF was performed a day after surgery on patients underwent ACLR with aggravated pain. Seven days follow up was worked up. The study was conducted in 2021. The inclusion criteria were: male, 18 to 40 years old, the patient who underwent isolated ACLR, using triple hamstring tendon graft, complained of severe pain a day after surgery, willingness to participate, and signing the informed consent form. The exclusion criteria were having a multi-ligament injury, involving in multiple surgery procedures, and using the addition of analgesic drug besides the drug in this protocol (ketorolac 30 mg intravenously in the first three days, and diclofenac sodium 50 mg twice a day orally after that).

**Fig. 1:**
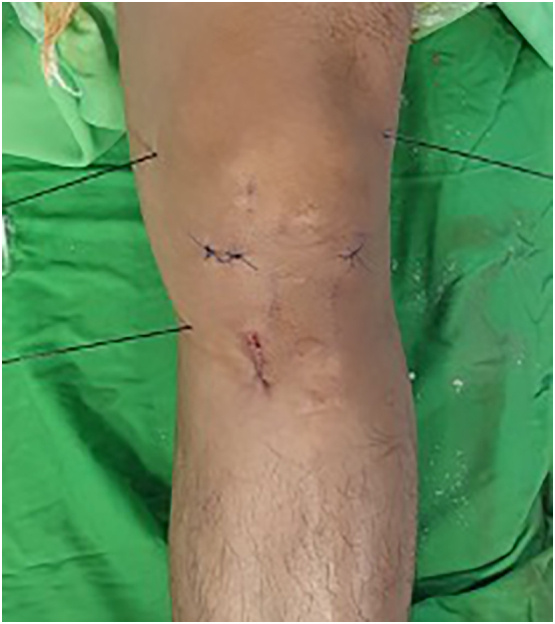
Place the cannula directed at the superior lateral genicular nerve, the superior medial genicular nerve, and the inferior medial genicular nerve.

**Fig. 2:**
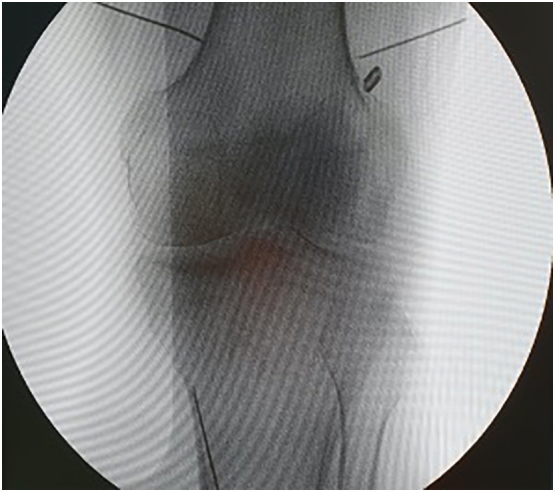
Fluoroscopic anteroposterior image after ultrasonography-guided needle insertion for genicular nerve block confirming placement of the cannula directed at the base of the medial and lateral femoral condyles and the base of the medial tibial.

GNRF was performed using RF device (Cosman RFG-4,United States). Patient in supine position with a pillow under the popliteal fossa to minimize discomfort. After applying the ground pad on the contralateral side and disinfecting the skin surrounding the affected site, we identify the three entry sites of the cannulas' insertion at the base of the medial and lateral femoral condyles and the base of the medial tibial condyle using ultrasonography (Wisonic Navi Color Doppler; China). Utilizing a 12-MHz linear transducer, we identify the superior lateral, superior medial, and inferior medial genicular artery and nerve. The location of the needle tip should be within the vicinity of each genicular artery. After identifying the genicular artery, the skin and subcutaneous tissue were anesthetized with 1 mL of 1% lidocaine at each target point. The needle was inserted using the long-axis view of the ultrasound probe. Next, we insert the cannulas with a 10-mm active tip percutaneously targeting the superior lateral genicular nerve, the superior medial genicular nerve, and the inferior medial genicular nerve (Fig. 1). We also confirmed the position by fluoroscopic anteroposterior image after ultrasonography-guided needle insertion (Fig. 2). Subsequently, sensory and motor testing was conducted. After proper placement, we injected 1 mL of 2% lidocaine before ablation through each cannula. Then, we give the pulsed RF in 42 °C for 3 min. Subsequently, we rotate the cannulas 180° and repeat the ablation for the next 3 min using the same settings. We remove the cannulas at the end of the procedures and apply a bandage.

All procedures were performed by a single orthopedic surgeon-pain interventionist (RD) experienced in pain intervention guided with ultrasonography and fluoroscopy. Patients received an analgesic drug (ketorolac 30 mg third a day intravenously in the first three days, followed by diclofenac sodium 50 mg twice a day after three days) during the study period.

The Visual Analogue Scale (VAS) score was used to evaluate the patients' pain during the follow-up study in the postoperative days (POD) first, third, and seventh. In addition, we also assessed the score of quality of life using Euroqol-5 Dimension (EQ-5D).

## Results

Two patients participated in the present study and completed the informed consent form. The subject's characteristics are presented in Table 1. The mean age of participants was 30.5 years, with the Tegner level of 5.

**Table 1:** Demographic patient characteristics (N = 106).

Characteristic	Value
Age (y), mean ± SD	30.5 ± 1.2
Affected side, n (%)	
Right	1 (50)
Left	1 (50)
Tegner level	5
Occupation, n (%)	
Athlete	0 (0)
Non-athlete	2 (100)
Activity at injury, n (%)	
ADL	0
Sport	2 (100)
Traffic	0
Work	0

Abbreviations: ACL—anterior cruciate ligament; y—years; ADL—activity of daily living; SD— standard deviation.

The mean of VAS pain scores and EQ5D were presented in Table 2. The mean of VAS pain scores in POD 1, 3, and 7 were 5, 2, and 1, respectively (VAS pre-GNRF was 8), and the mean of EQ5D was 0.52, 0.56, and 0.66, respectively (EQ5D pre-GNRF was 0.48).

**Table 2:** The mean of VAS pain scores and EQ5D pre and post-GNRF.

	POD 1 Before GNRF	POD 1 after GNRF	POD 3	POD 7
The mean of VAS	8	5	2	1
the mean of EQ5D	0.48	0.52	0.56	0.66

## Discussion

This study aimed to evaluate the use of GNRF in patients who underwent ACLR. This study found that the mean VAS pain score after GNRF was decreased, and the mean of EQ5D was improved. These results concluded that GNRF is an adequate and safe procedure for managing postoperative pain after ACLR, and it may enhance the postoperative rehabilitation program.

GNRF was known to give promising results for knee pain treatment. Numerous studies, however, yielded concerns about chronic knee pain, osteoarthritis, and post-total knee arthroplasty procedures [9], [10], [11], [12]. The GNRF can offer substantial clinical and functional benefits to patients with knee pain related. In other studies, GNRF has been described in the treatment of knee OA within the pericapsular nerve endings [13] and the sciatic nerve [14]. Long-term efficacy of pulsed RFA of the saphenous nerve has been demonstrated in treating chronic knee pain [15], and pulsed RFA of the entire nerve supply of the knee showed improved functional outcomes in patients with knee OA [16]. Recently, Carrier et al. [17] was found that GNRF was also effective in knee pain after trauma case. In this study, we performed GNRF for patients who underwent ACLR with aggravated pain, and we showed the efficacy of GNRF for these cases was promising.

In this study, we performed GNRF by ultrasonography-guided rather than fluoroscopy-guided only. A growing body of knowledge described that GNRF by ultrasonography-guided more accurate and safer than fluoroscopy guiding [18], [19], [20], [21]. However, Kim et al. [21] showed that no significant differences in pain relief, improvements in knee functionality, or safety were observed between the two methods. Therefore, both ultrasonography and fluoroscopy-guided might be selected for GNRF treatment. Nevertheless, we consider that ultrasonography-guided might be a better option for GNRF treatment because of radiation exposure issues and other benefits.

GNRF is a safe procedure with minimal complications [11], [20], [21]. Kim et al. (2016) [11] described that RF had a risk of iatrogenic side effects, especially the vascular injury in the formation of pseudoaneurysm, arteriovenous fistula, hemarthrosis, and osteonecrosis of the patella. However, we did not find this effect in our study. We use pulsed GNRF that relatively no tissue damage occurs as the temperature is set at a limit of 42 °C. It might explain the lack of side effects in our study.

As it still is a relatively advanced method, the information about the long-term efficacy and adverse events is still largely lacking. A commonly raised question is whether this procedure precipitates a Charcot-type joint in the long term [10], even though this assumption has lacked supporting data. To our knowledge, no Charcot-type joints have been reported after this procedure. However, further studies are needed to evaluate this treatment in an extended period.

The current study has limitations, including the sample size is not large enough, performed only in a single-centre study, and not comparing other treatments, such as femoral nerve block or adductor canal block, that are more familiar for managing postoperative pain after ACLR. Hence, the authors suggest performing multi-centre prospective studies in many patients and comparing the outcomes with other studies.

## Conclusions

GNRF is an adequate and safe procedure for managing postoperative pain after ACLR. It may enhance the postoperative rehabilitation program.
